# Sex/gender and socioeconomic differences in modifiable risk factors for dementia

**DOI:** 10.1038/s41598-022-27368-4

**Published:** 2023-01-03

**Authors:** Anouk F. J. Geraets, Anja K. Leist

**Affiliations:** Department of Social Sciences, University of Luxembourg, Campus Belval, Maison Des Sciences Humaines, 11 Porte Des Sciences, L-4366 Esch-Sur-Alzette, Luxembourg

**Keywords:** Risk factors, Disease prevention, Public health, Epidemiology, Dementia

## Abstract

Both sex/gender and socioeconomic differences have been reported in the prevalence of modifiable risk factors for dementia. However, it remains unclear whether the associations between modifiable risk factors for dementia and incident dementia differ by sex/gender or socioeconomic status. This study aimed to investigate sex/gender and socioeconomic differences in the associations of modifiable risk factors with incident dementia using a life-course perspective. We used data from the English Longitudinal Study of Ageing (2008/2009 to 2018/2019). A total of 8,941 individuals were included [mean (standard deviation) age, 66.1 ± 9.8 years; 4,935 (55.2%) were women]. No overall sex/gender difference in dementia risk was found. Dementia risk was higher among those who experienced childhood deprivation [hazard ratio (HR) = 1.51 (1.17; 1.96)], lower occupational attainment [HR low versus high = 1.60 (1.23; 2.09) and HR medium versus high = 1.53 (1.15; 2.06)], and low wealth [HR low versus high = 1.63 (1.26; 2.12)]. Though different associations were found among the subgroups, there might be a sex/gender difference in dementia risk only for low cognitive activity, suggesting a higher risk for women [HR = 2.61 (1.89; 3.60)] compared to men [HR = 1.73 (1.20; 2.49)]. No consistent socioeconomic differences in modifiable dementia risk were found. A population-based approach that tackles inequalities in dementia risk profiles directly may be more effective than individual approaches in dementia prevention.

## Introduction

Dementia is a major cause of disability and dependency among older people^1^. Currently more than 55 million people live with dementia, and this number is expected to rise to 139 million in 2050 worldwide^1^. Though the overall prevalence of dementia continues to increase, there is evidence for a decrease in dementia incidence in Western high-income countries^2,3^. This decrease in dementia incidence can be the result of improvements in modifiable risk factors for dementia, but it can also be the result of increases in cognitive reserve caused by increases in population levels of educational and occupational attainmen^2,3^. A total of 12 modifiable risk factors have been reported to contribute to up to 40% of all dementia cases^4^. These factors include cardiometabolic risk factors (hypertension, diabetes, smoking, obesity, high alcohol consumption, and physical inactivity), behavioral factors (depression, low educational attainment, low social contact), environmental factors (air pollution) and other conditions (hearing impairment and traumatic brain injury)^4^. Other potential modifiable risk factors for dementia include unhealthy diet, cognitive inactivity, sleep disturbance, hypercholesterolemia, coronary heart disease, and renal dysfunction^4,5^. Population-level evidence suggests strong, consistent, biologically plausible and dose–response relationships between these modifiable risk factors and dementia, even if not all risk factors have been established as causal determinants of dementia^6^.

Sex/gender differences in modifiable risk factors for dementia have been reported^7^. Though sex and gender are different concepts that may be differently related to cognition (biological versus psychosocial, respectively)^8^, we use the term sex/gender because we don’t know whether we measured sex or gender. For example, the prevalence of diabetes is higher among men^9^, while the prevalence of depression is higher among women^10^. However, it is unclear whether exposure-outcome relationships differ by sex/gender, or if risk factors contribute similarly to dementia risk. A study that included data from three large Nordic population-based cohort studies found that the association of risk factors for dementia with incident dementia may differ by sex/gender^11^. For instance, physical activity and education were associated with a lower risk for dementia among women but not among men. However, results for other dementia risk factors were inconsistent between short (3–10 years) and long (20–30 years) term follow-up, and the effects of a multidomain lifestyle intervention on cognitive change did not differ between men and women. Gong et al. found that smoking, diabetes, stroke and area deprivation were associated with a greater risk of dementia, similarly in women and men using data of the UK Biobank^12^. However, the association between systolic blood pressure and dementia was *U*-shaped in men but had a positive dose–response relationship in women. Furthermore, obesity was associated with an increased dementia risk among women, but a decreased dementia risk among men. Another UK Biobank study found that women with cardiovascular disease had a 20% increased risk for dementia compared to men with cardiovascular disease^13^.

The risk for dementia might also differ by socioeconomic status (SES), indicated by differences in education, income, wealth, or (parental) occupational status. Studies that used data from the English Longitudinal Study of Ageing (ELSA) have shown that lower wealth in late-life is associated with increased risk for dementia^14,15^. Furthermore, results from the Whitehall II study suggest that low midlife occupation was associated with an increased dementia risk^16^. Other ELSA research found that childhood socioeconomic disadvantages were linked to late-life cognitive performance^17^. Socioeconomic inequalities in dementia risk may be the result of differences in prevalence of modifiable risk factors for dementia^18,19^ but may also be the result of differences in opportunities for cognitively and socially stimulating activities and the social environment at large^20^. In this sense, they express differences in cognitive reserve^21^. While it is difficult to intervene on contextual socioeconomic conditions, the investigation of possible SES differences in modifiable risk factors for dementia is highly relevant, as these differences may be overcome through targeted population-based healthcare and prevention offers to more vulnerable individuals. Deckers et al. found that 52% of the increase dementia risk for individuals with low wealth compared to high wealth was mediated by differences in modifiable risk factors for dementia in ELSA^15^.

A better insight into modifiable risk factors for dementia using a life-course perspective may contribute to more tailored prevention strategies. SES assessed at different stages of the life course, specifically through deprivation and parental occupation during childhood, education attained in early adulthood, current or last occupation, and later-life wealth, may differentially modify the association of modifiable risk factors for dementia and incidence of dementia. While there may be sex/gender and SES differences in the prevalence of modifiable risk factors for dementia, it remains unclear whether the associations between modifiable risk factors for dementia and incident dementia differ by sex/gender or SES. This study aimed to investigate sex/gender and SES differences in the associations of modifiable vascular, metabolic and behavior factors with dementia risk using a life-course perspective.

## Methods

### Study population and design

Longitudinal data from the English Longitudinal Study of Ageing (ELSA) was used for this study. ELSA is a population-based cohort study among the English population aged 50 years and above^22^. Measures of SES, health, and behavior have been collected bi-annual from 2002/2003 (Wave 1) until 2018/2019 (Wave 9). More details have been described elsewhere^23^. The National Health Service Multicenter Research and Ethics Committee and the University College London Research Ethics Committee approved the study according to the guidelines of the Helsinki Declaration. All participants provided written informed consent.

Since Wave 4 (2008/2009; *n* = 9886) assessed many modifiable risk factors for dementia, it was considered as the baseline for this study. In case of missing information on SES, risk factors for dementia or potential confounders, non-missing data from adjacent waves [in most cases Wave 3 (2006/2007) or Wave 5 (2010/2011)] was used (Supplementary eTable 1). The last follow-up measurement was Wave 9 (2018/2019), yielding a maximum follow-up period of 11 years. Participants with prevalent dementia, missing data on all SES factors, unavailable data on modifiable risk factors, and unavailable data during follow-up were excluded from analyses. We used complete case analyses in which we included all participants with available data on the determinants and outcome under investigation.

### Childhood SES

Self-reported childhood deprivation and occupational attainment of the family breadwinner were included as measures of childhood SES. Childhood deprivation was assessed during Wave 3 of ELSA, in which questions about the home and family environments around 10 years of age were asked. Childhood deprivation (yes/no) was classified as having one or more of the following deprivations: (1) having none or few books (0–10 books) in the home; (2) overcrowding in the home (more than two persons per bedroom); and (3) having no indoor toilet, no fixed bath, no central heating, and/or no hot and cold running water supply to the home. Occupational attainment of the family breadwinner during childhood was classified using the International Labor Organization’s definition of skill level^24^ during Wave 1–9. Managers, senior officials, business owners, professionals, and technicians were classified as highly skilled. Response options “armed forces” “other jobs”, “something else” and “retired” were classified as missing due to the lack of clarity of the (previous) positions. All other categories were classified as lower-skilled.

### Adulthood SES

Self-reported occupational attainment and wealth were assessed as measures of SES during adulthood. Occupational attainment was based on the U.K. National Statistics Socioeconomic Classification (NS-SEC) system, which groups people based on working conditions, relations, and rewards^25^. We used the three-level schema as only this classification can be treated as having a hierarchical order^25^. These occupational levels are: (1) routine or manual; (2) intermediate; and (3) managerial or professional. Self-reported household wealth was calculated by summing wealth from the total value of a respondent’s home (minus outstanding mortgage payments), physical wealth (e.g., jewelry), business assets (e.g., investments), and financial assets such as cash and savings (minus debts and loans). The overall measure of wealth was divided into tertiles.

### Modifiable risk factors for dementia

Modifiable risk factors for dementia were selected based on a systematic review and Delphi consensus study^5^ and a Lancet Commission update on dementia prevention in 2020^4^. Individual exposure to modifiable risk factors for dementia was based on clinical data from nurse visits or self-reported information. Modifiable risk factors for dementia included heart disease, hypertension, hypercholesterolemia, diabetes, obesity, hearing impairment, smoking, high alcohol consumption, physical inactivity, sleep disturbance, unhealthy diet, depression, low educational attainment, low social contact, and low cognitive activity. Renal disease, air pollution, and traumatic brain injury, modifiable risk factors for dementia reported in the systematic review and Delphi consensus study^5^ and Lancet update 2020^4^, were not available in ELSA. Self-reported educational attainment was regrouped into two categories: low (no formal qualifications) and medium/high (National Vocational Qualifications level 1 till degree level). Participants with a foreign or other educational qualification were classified as missing due to the lack of clarity of the qualification. More information about the operationalization of the dementia risk factors can be found in the Supplemental Material (eTable 2).

### Dementia

Incidence of dementia was assessed at each wave by use of a combined algorithm of (1) self-reported or informant-reported physician-diagnosis of dementia or Alzheimer’s disease; or (2) an average score of ≥ 3.38 on the 16-question the Informant Questionnaire on Cognitive Decline in the Elderly (IQCODE)^26–29^. This questionnaire uses informant reports to evaluate the changes in every day cognitive function (e.g., memory) since the last interview^30^. Each item was scored on a 1 (much improved) to 5 (much worse) range. The validity of this scale was previously examined, and the threshold used has both high specificity (0.84) and sensitivity (0.82)^27,29^.

### Statistical analyses

Analyses were conducted in Stata 17.0 (StataCorp, College Station, TX, USA). Cox proportional hazard regressions were used to assess the associations between modifiable risk factors for dementia and time to dementia, resulting in hazard ratios (HR) and their 95% confidence interval (CI). The failure event was incident dementia. Lapse in time in years was used as the time axis, with date of birth as origin, individuals entering the risk set at the date of Wave 4 assessment and exiting (end of survival time) at date of dementia diagnosis (as reported by the participant or calculated as the mid-point between waves) or study exit (date of death or date at the last interview), whichever came first. Since participants could come from the same household, we used the Huber-White sandwich estimator to allow clustering at the household level^31^. In addition, a sampling weight (baseline cross-sectional weight) was used to back-weight estimates from the analysis sample to the total sample to minimize selection bias. Analyses in the total study sample were adjusted for age, sex/gender, wealth, and clustering at the household level. Sex/gender-specific analyses were adjusted for age, wealth, and clustering at the household level, and SES-specific analyses were adjusted for age, sex/gender, and clustering at the household level. To test whether the association between the modifiable risk factors and incident dementia differed by sex, we tested the interaction of the modifiable risk factors with sex/gender on incident dementia with men as the reference category. Differences between the risk factors and incident dementia by SES were tested with the interaction of the risk factors with SES on incident dementia with respectively high childhood SES and high adulthood SES as reference categories. A two-sided *p* value of 0.05 was applied.

### Sensitivity analyses

To evaluate differences between participants included and excluded in the analyses, we tested for differences in demographics, SES, risk factors for dementia, and incident dementia using independent *t* tests, Mann–Whitney *U* tests or *χ*^2^ tests. Because previous studies only found on increased dementia risk among women for those aged > 80 years^11^, we assessed overall sex/gender differences in dementia risk in subpopulations aged ≤ 80 years and aged > 80 years. Lastly, we investigated multiple interactions. Correction for multiple testing reduces the chance of type 1 error at the cost of increasing the risk for type 2 error^32^. Because our study is explorative in nature, we corrected for multiple testing in sensitivity analyses in which *p* values for the interactions were adjusted according to the Benjamini–Hochberg procedure^33^. This procedure controls the proportion of false positives instead of the probability of a single false-positive among all tests. The False Discovery Rate was set at 0.20, meaning that 20% of the associations found in our analyses are expected not to be true.

## Results

### Baseline characteristics

**Figure 1:**
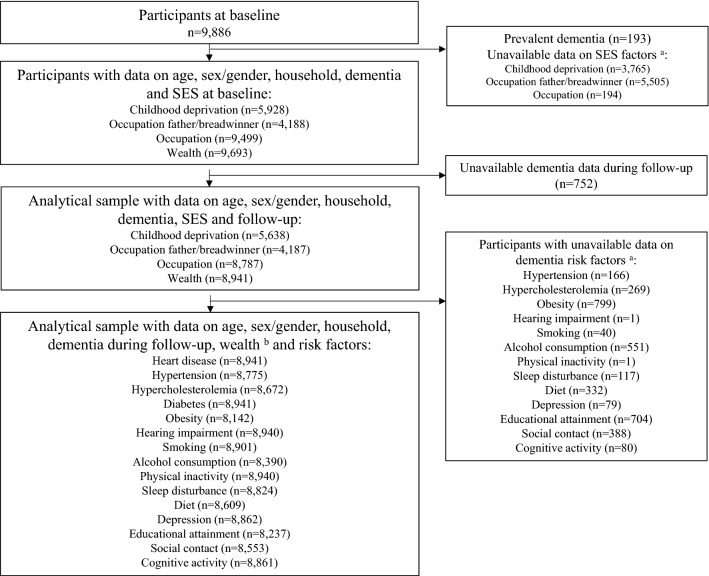
Flowchart. (**a**) Missing data are not mutually exclusive. (**b**) Data on childhood deprivation, occupation breadwinner and occupation are missing in respectively *n* = 3303, *n* = 4754, and *n* = 154 participants.

Figure 1 shows the flowchart of the study population. From the initial 9,886 participants, 193 participants had prevalent dementia and *n* = 3765, *n* = 5505, and *n* = 194 had missing data on respectively childhood deprivation, occupation of breadwinner, and occupation during adulthood. Another 752 participants did not have information on dementia status during follow-up. Our analytical sample ranged between *n* = 8941 and *n* = 5272, depending on the socioeconomic indicator and risk factor included in the model (e.g., *n* = 8941 for sex and diabetes and *n* = 5272 for childhood SES and obesity).

During 70,564 person-years of follow-up, 396 participants (4.4%) developed dementia (*n* = 340 based on self-reported diagnosis and *n* = 56 additional based on the IQCODE). Table 1 shows the characteristics of the study sample at baseline, stratified for incident dementia. Participants had a mean age of 66.1 ± 9.8 years and 55.2% were women. Participants with incident dementia were older, more deprived during childhood, and had a lower SES during adulthood. Furthermore, they had a worse dementia risk profile.

**Table 1:** Characteristics study population.

	Incident dementia	
	No (*n* = 8545)	Yes (*n* = 396)
**Demographics**		
Age, mean (standard deviation)	65.6 (9.6)	75.4 (9.2)
Women, *n* (%)	4693 (54.9)	242 (61.1)
**Socioeconomic status**		
Childhood deprivation, *n* (%)	2190 (40.7)	135 (53.6)
Low/medium occupation breadwinner, *n* (%)	2551 (63.0)	91 (66.9)
**Occupation, *n* (%)**		
Low	3327 (39.6)	181 (47.1)
Medium	2140 (25.5)	111 (28.9)
High	2936 (34.9)	92 (34.5)
**Wealth, *n* (%)**		
Low	2763 (32.3)	167 (42.2)
Medium	2918 (34.2)	129 (32.6)
High	2864 (33.5)	100 (25.3)
**Dementia risk factors**		
Heart disease, *n* (%)	879 (10.3)	77 (19.4)
Hypertension, *n* (%)	5338 (63.7)	304 (77.6)
Hypercholesterolemia, *n* (%)	4867 (58.7)	217 (56.7)
Diabetes, *n* (%)	2037 (23.8)	120 (30.3)
Obesity, *n* (%)	2563 (32.9)	105 (30.2)
Hearing impairment, *n* (%)	1646 (19.3)	134 (33.8)
Smoking, *n* (%)	1181 (13.9)	42 (10.7)
High alcohol consumption, *n* (%)	3626 (45.1)	194 (56.2)
Physical inactivity, *n* (%)	2448 (28.7)	201 (50.8)
Sleep disturbance, *n* (%)	4738 (56.1)	227 (59.3)
Unhealthy diet, *n* (%)	3364 (40.8)	161 (43.9)
Depression, *n* (%)	1762 (20.8)	121 (31.0)
Low education, *n* (%)	2382 (30.3)	175 (48.3)
Low social contact, *n* (%)	2410 (29.4)	126 (34.7)
Low cognitive activity, *n* (%)	2621 (31.0)	238 (60.6)

Characteristics of the study population stratified by sex/gender and wealth are shown in Supplementary eTables 3 and 4. Women more often had incident dementia, were older, had a lower adulthood SES, more often had hypercholesterolemia, obesity, high alcohol consumption, physical inactivity, and depression, and had a lower education, social contact and cognitive activity compared to men, while men more often had hypertension, diabetes, hearing impairment, and an unhealthy diet compared to women (Supplementary eTable 3). Regarding SES, participants with lower wealth had a worse dementia risk profile (Supplementary eTable 4). All modifiable risk factors were more prevalent in individuals with lower wealth, apart from hypercholesteremia that showed an inverse relation and was more prevalent in individuals reporting higher wealth.

### Sex/gender difference in dementia risk

No overall sex/gender difference in dementia risk was found [HR women versus men = 1.08 (0.87; 1.33), *p* = 0.487] after adjustment for age, wealth and clustering at the household level. Table 2 shows the total and sex/gender-specific associations of the dementia risk factors with incident dementia and the interactions of the dementia risk factors with sex/gender on incident dementia. In the total population, diabetes [HR = 1.41 (1.11; 1.78), *p* = 0.004], hearing impairment [HR = 1.43 (1.14; 1.79), *p* = 0.002], high alcohol consumption [HR = 1.27 (1.02; 1.60), *p* = 0.036], physical inactivity [HR = 1.67 (1.32; 2.10), *p* < 0.001], depression [HR = 1.64 (1.28; 2.09), *p* < 0.001], low education [HR = 1.29 (1.02; 1.63), *p* = 0.036], low social contact [HR = 1.44 (1.15; 1.80), *p* = 0.002], and low cognitive activity [HR = 2.17 (1.71; 2.77), *p* < 0.001] were associated with an increased risk for dementia.

**Table 2:** The total and sex/gender-specific associations of dementia risk factors with incident dementia and the interactions of the dementia risk factors with sex on dementia risk.

Dementia risk factors	Total (in = 8941)		Women (*n* = 4935)		Men (*n* = 4006)		Interaction^a^ (*n* = 8941)	
	**Incident dementia (*n* = 396) HR (95% CI)**	***p* value**	**Incident dementia (in = 242) HR (95% CI)**	***p* value**	**Incident dementia (*n* = 154) HR (95% CI)**	***p* value**	**Incident dementia (*n* = 396) HR (95% CI)**	***p* value**
Heart disease	1.20 (0.93; 1.57)	0.166	0.97 (0.66; 1.41)	0.859	**1.53 (1.05; 2.24)**	**0.028**	0.69 (0.41; 1.17)	0.167
Hypertension	1.29 (0.99; 1.68)	0.055	1.28 (0.89; 1.82)	0.178	1.26 (0.86; 1.84)	0.244	1.11 (0.67; 1.85)	0.689
Hypercholesterolemia	0.93 (0.75; 1.15)	0.489	0.81 (0.61; 1.06)	0.125	1.13 (0.80; 1.58)	0.493	0.68 (0.44; 1.05)	0.080
Diabetes	**1.41 (1.11; 1.78)**	**0.004**	**1.36 (1.02; 1.83)**	**0.038**	**1.48 (1.02; 2.15)**	**0.038**	0.94 (0.59; 1.50)	0.798
Obesity	0.94 (0.74; 1.20)	0.643	0.95 (0.70; 1.28)	0.719	0.94 (0.62; 1.41)	0.762	1.03 (0.62; 1.72)	0.906
Hearing impairment	**1.43 (1.14; 1.79)**	**0.002**	1.26 (0.93; 1.71)	0.141	**1.69 (1.20; 2.37)**	**0.003**	0.80 (0.51; 1.26)	0.335
Smoking	1.38 (0.97; 1.92)	0.071	**1.63 (1.08; 2.46)**	**0.021**	0.99 (0.54; 1.82)	0.984	1.44 (0.70; 2.94)	0.320
High alcohol consumption	**1.27 (1.02; 1.60)**	**0.036**	**1.43 (1.04; 1.95)**	**0.027**	1.09 (0.77; 1.55)	0.607	1.29 (0.82; 2.04)	0.270
Physical inactivity	**1.67 (1.32; 2.10)**	** < 0.001**	**1.65 (1.25; 2.19)**	** < 0.001**	**1.69 (1.17; 2.44)**	**0.005**	1.09 (0.70; 1.67)	0.695
Sleep disturbance	0.98 (0.79; 1.22)	0.888	1.07 (0.81; 1.41)	0.643	0.91 (0.64; 1.28)	0.574	1.24 (0.80; 1.92)	0.336
Unhealthy diet	1.15 (0.92; 1.43)	0.211	1.19 (0.90; 1.57)	0.232	1.05 (0.75; 1.48)	0.772	1.11 (0.71; 1.72)	0.650
Depression	**1.64 (1.28; 2.09)**	** < 0.001**	**1.57 (1.19; 2.08)**	**0.001**	**1.83 (1.17; 2.85)**	**0.008**	0.90 (0.54; 1.50)	0.684
Low education	**1.29 (1.02; 1.63)**	**0.036**	1.20 (0.90; 1.62)	0.218	1.40 (0.96; 2.05)	0.081	1.32 (0.92; 1.90)	0.138
Low social contact	**1.44 (1.15; 1.80)**	**0.002**	**1.47 (1.09; 1.97)**	**0.011**	**1.45 (1.01; 2.08)**	**0.041**	1.00 (0.63; 1.59)	0.996
Low cognitive activity	**2.17 (1.71; 2.77)**	** < 0.001**	**2.61 (1.89; 3.60)**	** < 0.001**	**1.73 (1.20; 2.49)**	**0.003**	**1.57 (1.02; 2.41)**	**0.041**

*HR* hazard ratio; *CI* confidence interval. Analyses are adjusted for age, sex/gender (total population), wealth and clustering at the household level. ^a^The interaction of the risk factors with sex/gender on incident dementia with men as the reference category. Significant values are in bold.

No sex/gender differences in the associations between the risk factors and incident dementia were found aside for low cognitive activity (*p* interaction = 0.041), which suggested a stronger association between low cognitive activity and incident dementia among women [HR = 2.61 (1.89; 3.60), *p* < 0.001] compared to men [HR = 1.73 (1.20; 2.49), *p* = 0.003]. The association of hearing impairment with incident dementia attenuated and became statistically non-significant among women [HR = 1.26 (0.93; 1.71), *p* = 0.141] and the association of high alcohol consumption with incident dementia attenuated and became statistically non-significant among men [HR = 1.09 (0.77;1.55), *p* = 0.607]. The association of low education with incident dementia became statistically non-significant among both sexes/genders [HR = 1.20 (0.90; 1.62), *p* = 0.218 for women and HR = 1.40 (0.96; 2.05), *p* = 0.081 for men; Table 2].

### Childhood SES differences in dementia risk

Individuals who experienced childhood deprivation had a higher dementia risk compared to those without a history of childhood deprivation [HR = 1.51 (1.17; 1.96), *p* = 0.001] after adjustment for age, sex/gender, and clustering at the household level. No overall difference in dementia risk was found between individuals whose breadwinner during childhood had a low/medium occupation versus a high occupation [HR = 1.08 (0.75; 1.57), *p* = 0.676].

Table 3 shows the associations of the dementia risk factors with incident dementia stratified by childhood SES and the interactions of the dementia risk factors with childhood SES on dementia risk. In stratified analyses, heart disease, diabetes, hearing impairment, smoking, physical inactivity, depression, and low cognitive activity were associated with incident dementia among those with a low/medium SES during childhood. Among participants with a high SES during childhood, heart disease, smoking, physical inactivity, low social contact, and low cognitive activity were associated with an increased risk for dementia. One interaction of childhood deprivation with heart disease on incident dementia was found (*p* interaction = 0.014), suggesting an association between heart disease and incident dementia for individuals without childhood deprivation [HR = 1.99 (1.25; 3.16), *p* = 0.004] but not for those who experienced childhood deprivation [HR = 0.87 (0.54; 1.39), *p* = 0.550]. No other interactions of childhood SES with dementia risk factors on incident dementia were found.

**Table 3:** The association of dementia risk factors with incident dementia stratified by indicators of childhood socioeconomic status (childhood deprivation yes and no; occupation breadwinner low/medium and high) and the interactions of the dementia risk factors with childhood socioeconomic status on dementia risk.

Dementia risk factors	Childhood SES: Low/medium		Childhood SES: High		Interaction^a^	
	Incident dementia HR (95% CI)	*p* value	Incident dementia HR (95% CI)	*p* value	Incident dementia HR (95% CI)	*p* value
**Heart disease**						
Childhood deprivation	0.87 (0.54; 1.39)	0.550	**1.99 (1.25; 3.16)**	**0.004**	**0.45 (0.24; 0.85)**	**0.014**
Occupation breadwinner	**2.25 (1.34; 3.76)**	**0.002**	1.21 (0.48; 3.07)	0.682	1.39 (0.50; 3.89)	0.527
**Hypertension**						
Childhood deprivation	1.11 (0.70; 1.78)	0.653	1.50 (0.96; 2.35)	0.073	0.71 (0.38; 1.34)	0.298
Occupation breadwinner	1.47 (0.89; 2.41)	0.133	2.00 (0.99; 4.05)	0.053	0.61 (0.26; 1.44)	0.258
**Hypercholesterolemia**						
Childhood deprivation	0.92 (0.64; 1.32)	0.640	1.16 (0.77; 1.75)	0.471	0.76 (0.45; 1.31)	0.328
Occupation breadwinner	0.91 (0.58; 1.44)	0.686	0.59 (0.32; 1.09)	0.093	1.49 (0.71; 3.13)	0.296
**Diabetes**						
Childhood deprivation	1.33 (0.89; 2.00)	0.164	0.99 (0.60; 1.65)	0.982	1.34 (0.70; 2.55)	0.378
Occupation breadwinner	**1.90 (1.15; 3.13)**	**0.012**	1.17 (0.54; 2.55)	0.687	1.55 (0.63; 3.80)	0.340
**Obesity**						
Childhood deprivation	0.93 (0.62; 1.40)	0.738	0.78 (0.50; 1.21)	0.271	1.19 (0.66; 2.16)	0.562
Occupation breadwinner	0.76 (0.45; 1.26)	0.286	0.81 (0.38; 1.71)	0.576	0.98 (0.39; 2.44)	0.966
**Hearing impairment**						
Childhood deprivation	**1.60 (1.11; 2.29)**	**0.011**	1.23 (0.77; 1.98)	0.388	1.28 (0.73; 2.25)	0.394
Occupation breadwinner	**1.87 (1.17; 2.98)**	**0.009**	1.72 (0.85; 3.47)	0.131	0.99 (0.45; 2.21)	0.989
**Smoking**						
Childhood deprivation	1.35 (0.79; 2.31)	0.270	**2.11 (1.16; 3.84)**	**0.014**	0.70 (0.32; 1.56)	0.383
Occupation breadwinner	**1.93 (1.09; 3.43)**	**0.025**	1.48 (0.45; 4.88)	0.523	1.83 (0.48; 6.93)	0.376
**High alcohol consumption**						
Childhood deprivation	1.40 (0.96; 2.06)	0.084	1.22 (0.81; 1.83)	0.346	1.16 (0.67; 2.03)	0.592
Occupation breadwinner	1.19 (0.75; 1.89)	0.450	1.48 (0.81; 2.71)	0.205	0.80 (0.37; 1.71)	0.558
**Physical inactivity**						
Childhood deprivation	**1.61 (1.12; 2.31)**	**0.010**	**1.55 (1.02; 2.35)**	**0.042**	0.98 (0.58; 1.65)	0.930
Occupation breadwinner	**1.85 (1.14; 2.99)**	**0.013**	1.35 (0.72; 2.54)	0.346	1.03 (0.48; 2.24)	0.936
**Sleep disturbance**						
Childhood deprivation	0.83 (0.57; 1.20)	0.315	1.05 (0.71; 1.55)	0.804	0.78 (0.46; 1.32)	0.347
Occupation breadwinner	1.06 (0.66; 1.68)	0.816	1.26 (0.70; 2.29)	0.442	0.76 (0.37; 1.59)	0.469
**Unhealthy diet**						
Childhood deprivation	1.26 (0.87; 1.82)	0.219	1.38 (0.93; 2.03)	0.110	0.93 (0.54; 1.59)	0.783
Occupation breadwinner	1.18 (0.75; 1.87)	0.469	1.09 (0.55; 2.16)	0.799	1.21 (0.54; 2.70)	0.647
**Depression**						
Childhood deprivation	1.24 (0.81; 1.91)	0.318	1.30 (0.79; 2.12)	0.301	0.92 (0.49; 1.72)	0.791
Occupation breadwinner	**2.25 (1.31; 3.86)**	**0.003**	1.65 (0.79; 3.48)	0.185	1.24 (0.52; 2.98)	0.624
**Low education**						
Childhood deprivation	1.22 (0.81; 1.84)	0.340	1.45 (0.94; 2.23)	0.089	0.79 (0.45; 1.38)	0.408
Occupation breadwinner	1.40 (0.84; 2.30)	0.193	1.78 (0.87; 3.64)	0.113	0.59 (0.26; 1.32)	0.198
**Low social contact**						
Childhood deprivation	1.20 (0.81; 1.76)	0.359	1.26 (0.81; 1.95)	0.309	0.97 (0.54; 1.75)	0.918
Occupation breadwinner	1.55 (0.97; 2.47)	0.068	**2.36 (1.17; 4.77)**	**0.017**	0.77 (0.33; 1.80)	0.549
**Low cognitive activity**						
Childhood deprivation	**1.81 (1.23; 2.67)**	**0.003**	**2.93 (1.93; 4.45)**	** < 0.001**	0.64 (0.38; 1.08)	0.097
Occupation breadwinner	**2.79 (1.77; 4.39)**	** < 0.001**	**2.93 (1.58; 5.42)**	**0.001**	0.73 (0.35; 1.52)	0.396

Childhood deprivation *n* = 2325 (135 dementia cases) and no childhood deprivation *n* = 3313 (117 dementia cases). Low/medium occupation breadwinner *n* = 2642 (91 dementia cases) and high occupation breadwinner *n* = 1545 (45 dementia cases). *SES* socioeconomic status; *HR* hazard ratio; *CI* confidence interval. Analyses are adjusted for age, sex/gender, and clustering at the household level. ^a^The interaction of the risk factors with childhood SES on incident dementia with high childhood SES as the reference category. Significant values are in bold.

### Adulthood SES differences in dementia risk

Individuals with both a low and medium occupational attainment had an increased dementia risk compared to those with a high occupational attainment [HR = 1.60 (1.23; 2.09), *p* < 0.001 and HR = 1.53 (1.15; 2.06), *p* = 0.004, respectively] after adjustment for age, sex/gender, and clustering at the household level. Regarding wealth, we only found an overall difference in dementia risk between participants with low and high wealth [HR = 1.63 (1.26; 2.12), *p* < 0.001] but not between those with a medium and high wealth [HR = 1.22 (0.93; 1.60), *p* = 0.151] after adjustment for age, sex, and clustering at the household level.

Stratified analyses by adulthood SES are shown in Table 4. Among individuals with a low adulthood SES, hearing impairment, physical inactivity, depression, low education, low social contact, and low cognitive activity were associated with an increased risk for dementia. Among those with a medium adulthood SES, diabetes, physical inactivity, depression, and low cognitive activity were associated with increased dementia risk, while in those with a high adulthood SES, heart disease, hypertension, high alcohol consumption, physical inactivity, depression, and low cognitive activity were associated with incident dementia. Though associations differed among the SES groups, only one interaction of adulthood SES with the risk factors on incident dementia was found, suggesting an association between heart disease and incident dementia for individuals with a high occupation [HR = 1.24 (1.36; 3.68), *p* = 0.002] but not for those with a low occupation [HR = 0.83 (0.54; 1.26), *p* = 0.380; *p* interaction = 0.001]. No other interactions of adulthood SES with dementia risk factors on incident dementia were found (Table 5).

**Table 4:** The association of dementia risk factors with incident dementia stratified by indicators of adulthood socioeconomic status (occupation low/medium/high; wealth low/medium/high).

Dementia risk factors	Low adulthood SES		Medium adulthood SES		High adulthood SES	
	Incident dementia HR (95% CI)	*p* value	Incident dementia HR (95% CI)	*p* value	Incident dementia HR (95% CI)	*p* value
**Heart disease**						
Occupation ^a^	0.83 (0.54; 1.26)	0.380	1.45 (0.87; 2.41)	0.157	**2.24 (1.36; 3.68)**	**0.002**
Wealth	1.10 (0.74; 1.63)	0.648	1.13 (0.70; 1.82)	0.615	1.60 (0.94; 2.71)	0.083
**Hypertension**						
Occupation ^a^	1.02 (0.72; 1.47)	0.897	1.62 (0.98; 2.69)	0.062	**1.85 (1.08; 3.16)**	**0.025**
Wealth	1.40 (0.88; 2.22)	0.160	1.22 (0.78; 1.90)	0.381	1.24 (0.79; 1.96)	0.352
**Hypercholesterolemia**						
Occupation ^a^	0.84 (0.62; 1.15)	0.288	1.06 (0.71; 1.58)	0.781	1.04 (0.66; 1.64)	0.859
Wealth	1.03 (0.74; 1.44)	0.845	0.79 (0.54; 1.16)	0.224	0.94 (0.61; 1.45)	0.789
**Diabetes**						
Occupation ^a^	1.31 (0.94; 1.82)	0.113	**1.59 (1.04; 2.45)**	**0.033**	1.20 (0.69; 2.08)	0.528
Wealth	1.29 (0.92; 1.80)	0.140	**1.62 (1.08; 2.42)**	**0.019**	1.50 (0.90; 2.48)	0.116
**Obesity**						
Occupation ^a^	0.89 (0.63; 1.26)	0.517	0.95 (0.60; 1.52)	0.831	1.18 (0.70; 1.97)	0.539
Wealth	0.93 (0.65; 1.32)	0.681	1.07 (0.70; 1.65)	0.750	0.79 (0.47; 1.32)	0.368
**Hearing impairment**						
Occupation ^a^	**1.57 (1.15; 2.15)**	**0.005**	1.25 (0.80; 1.96)	0.321	1.49 (0.91; 2.45)	0.112
Wealth	1.39 (0.98; 1.95)	0.061	1.48 (0.99; 2.20)	0.053	1.48 (0.93; 2.36)	0.095
**Smoking**						
Occupation ^a^	1.43 (0.92; 2.21)	0.108	1.11 (0.53; 2.34)	0.776	2.11 (0.95; 4.70)	0.067
Wealth	1.30 (0.84; 2.02)	0.245	1.51 (0.77; 2.97)	0.233	1.36 (0.55; 3.33)	0.501
**High alcohol consumption**						
Occupation ^a^	1.35 (0.97; 1.89)	0.078	1.40 (0.92; 2.12)	0.116	1.05 (0.67; 1.63)	0.844
Wealth	1.21 (0.83; 1.74)	0.321	1.04 (0.71; 1.52)	0.831	**1.78 (1.16; 2.72)**	**0.009**
**Physical inactivity**						
Occupation ^a^	**1.56 (1.13; 2.15)**	**0.007**	**1.82 (1.19; 2.80)**	**0.006**	**2.07 (1.25; 3.43)**	**0.005**
Wealth	**1.69 (1.20; 2.39)**	**0.003**	**2.15 (1.44; 3.21)**	** < 0.001**	1.13 (0.68; 1.88)	0.647
**Sleep disturbance**						
Occupation ^a^	1.06 (0.77; 1.45)	0.723	1.08 (0.72; 1.62)	0.723	0.90 (0.57; 1.43)	0.650
Wealth	1.16 (0.83; 1.63)	0.382	0.90 (0.61; 1.31)	0.576	0.85 (0.56; 1.30)	0.458
**Unhealthy diet**						
Occupation ^a^	1.46 (0.98; 2.17)	0.060	0.78 (0.44; 1.37)	0.387	1.08 (0.61; 1.90)	0.795
Wealth	1.17 (0.77; 1.78)	0.458	1.17 (0.72; 1.91)	0.524	1.19 (0.71; 2.01)	0.514
**Depression**						
Occupation ^a^	**1.77 (1.27; 2.47)**	**0.001**	**1.75 (1.13; 2.73)**	**0.013**	1.41 (0.74; 2.68)	0.300
Wealth	**1.57 (1.12; 2.19)**	**0.008**	1.54 (0.94; 2.53)	0.085	**1.94 (1.18; 3.19)**	**0.009**
**Low education**						
Occupation ^a^	**1.56 (1.08; 2.26)**	**0.019**	1.00 (0.64; 1.55)	0.987	0.91 (0.48; 1.71)	0.766
Wealth	1.29 (0.90; 1.84)	0.164	1.45 (0.97; 2.17)	0.073	1.11 (0.64; 1.92)	0.715
**Low social contact**						
Occupation ^a^	**1.50 (1.09; 2.07)**	**0.013**	1.45 (0.96; 2.19)	0.076	1.22 (0.71; 2.10)	0.466
Wealth	**1.73 (1.23; 2.42)**	**0.002**	1.17 (0.76; 1.78)	0.477	1.24 (0.73; 2.12)	0.426
**Low cognitive activity**						
Occupation ^a^	**2.05 (1.45; 2.90)**	** < 0.001**	**2.80 (1.80; 4.35)**	** < 0.001**	**1.59 (1.01; 2.51)**	**0.046**
Wealth	**1.87 (1.28; 2.75)**	**0.001**	**2.04 (1.39; 3.01)**	** < 0.001**	**2.95 (1.90; 4.59)**	** < 0.001**

Low occupation *n* = 3508 (181 dementia cases), medium occupation *n* = 2251 (111 dementia cases), and high occupation *n* = 3028 (92 dementia cases). Low wealth *n* = 2930 (167 dementia cases), medium wealth *n* = 3047 (129 dementia cases), and high wealth *n* = 2964 (100 dementia cases). *SES* socioeconomic status; *HR* hazard ratio; *CI* confidence interval. Analyses are adjusted for age, sex/gender, and clustering at the household level. ^a^Data missing in *n* = 151. Significant values are in bold.

**Table 5:** Interactions of dementia risk factors with indicators of adulthood socioeconomic status (occupation low/medium/high; wealth low/medium/high) on dementia risk.

Dementia risk factors	Low adulthood SES		Medium adulthood SES	
	Incident dementia HR (95% CI)	*p* value interaction^a^	Incident dementia HR (95% CI)	*p* value interaction^a^
**Heart disease**				
Occupation ^b^	**0.37 (0.20; 0.68)**	**0.001**	0.63 (0.32; 1.25)	0.188
Wealth	0.66 (0.35; 1.24)	0.194	0.67 (0.34; 1.34)	0.263
**Hypertension**				
Occupation ^b^	0.56 (0.30; 1.05)	0.072	0.87 (0.43; 1.76)	0.689
Wealth	1.10 (0.58; 2.10)	0.773	0.95 (0.51; 1.76)	0.864
**Hypercholesterolemia**				
Occupation ^b^	0.84 (0.49; 1.44)	0.522	1.03 (0.57; 1.85)	0.927
Wealth	1.14 (0.67; 1.95)	0.636	0.84 (0.48; 1.48)	0.550
**Diabetes**				
Occupation ^b^	1.06 (0.55; 2.04)	0.856	1.31 (0.65; 2.65)	0.450
Wealth	0.87 (0.48; 1.59)	0.650	1.03 (0.54; 1.95)	0.929
**Obesity**				
Occupation ^b^	0.76 (0.41; 1.40)	0.381	0.80 (0.40; 1.60)	0.526
Wealth	1.21 (0.65; 2.24)	0.545	1.32 (0.68; 2.57)	0.404
**Hearing impairment**				
Occupation ^b^	1.02 (0.58; 1.79)	0.946	0.85 (0.45; 1.62)	0.619
Wealth	0.91 (0.52; 1.58)	0.735	0.94 (0.52; 1.71)	0.833
**Smoking**				
Occupation ^b^	0.67 (0.27; 1.69)	0.397	0.53 (0.18; 1.61)	0.263
Wealth	0.95 (0.35; 2.58)	0.925	1.12 (0.36; 3.42)	0.847
**High alcohol consumption**				
Occupation ^b^	1.28 (0.73; 2.24)	0.392	1.30 (0.71; 2.37)	0.391
Wealth	0.68 (0.38; 1.20)	0.181	0.56 (0.32; 1.00)	0.051
**Physical inactivity**				
Occupation ^b^	0.77 (0.45; 1.33)	0.357	0.85 (0.47; 1.55)	0.598
Wealth	1.39 (0.79; 2.45)	0.253	1.69 (0.94; 3.05)	0.082
**Sleep disturbance**				
Occupation ^b^	1.17 (0.68; 2.01)	0.567	1.19 (0.65; 2.17)	0.572
Wealth	1.32 (0.77; 2.26)	0.308	1.05 (0.60; 1.84)	0.852
**Unhealthy diet**				
Occupation ^b^	1.53 (0.87; 2.69)	0.142	0.66 (0.35; 1.25)	0.203
Wealth	1.10 (0.63; 1.93)	0.742	1.35 (0.75; 2.41)	0.319
**Depression**				
Occupation ^b^	1.27 (0.65; 2.49)	0.482	1.19 (0.57; 2.47)	0.645
Wealth	1.10 (0.59; 2.06)	0.762	1.18 (0.61; 2.27)	0.620
**Low education**				
Occupation ^b^	1.70 (0.82; 3.53)	0.156	1.09 (0.50; 2.34)	0.785
Wealth	0.97 (0.56; 1.69)	0.920	0.95 (0.54; 1.67)	0.865
**Low social contact**				
Occupation ^b^	1.18 (0.63; 2.22)	0.607	1.18 (0.60; 2.34)	0.635
Wealth	1.37 (0.73; 2.59)	0.325	0.91 (0.46; 1.80)	0.784
**Low cognitive activity**				
Occupation ^b^	1.27 (0.73; 2.20)	0.399	1.54 (0.86; 2.77)	0.148
Wealth	0.65 (0.38; 1.12)	0.124	0.68 (0.39; 1.18)	0.171

Low occupation *n* = 3508 (181 dementia cases) and medium occupation *n* = 2251 (111 dementia cases). Low wealth *n* = 2930 (167 dementia cases) and medium wealth *n* = 3047 (129 dementia cases). *SES* socioeconomic status; *HR* hazard ratio; *CI* confidence interval. Analyses are adjusted for age, sex/gender, and clustering at the household level. ^a^Interaction of risk factor with adulthood SES with high adulthood SES as reference category (*n* = 2964 for wealth [100 dementia cases] and *n* = 3028 for occupation [92 dementia cases]). ^b^Data missing in *n* = 151. Significant values are in bold.

### Sensitivity analyses

Participants with incomplete data (*n* = 752) were more deprived during childhood, had a lower adulthood SES, and a worse dementia risk profile compared to participants included in the analytical sample (Supplementary eTable 5). Regarding overall sex/gender difference in dementia risk, no overall sex/gender difference in dementia risk was found in subpopulations aged ≤ 80 years [HR = 1.00 (0.78; 1.28), *p* = 0.994] or aged > 80 years [HR = 1.32 (0.86; 2.02), *p* = 0.209] after adjustment for age, wealth and clustering at the household level. According to the Benjamini–Hochberg procedure to correct for multiple testing, a 2-sided *P* value < 0.014 was considered statistically significant. After applying this correction for multiple testing, only the interaction of low occupation with heart disease on dementia risk remained significant, while the interactions of childhood deprivation with heart disease and sex/gender with low cognitive activity on dementia risk became non-significant.

## Discussion

This study aimed to investigate sex/gender and SES differences in the associations of modifiable vascular, metabolic and behavioral risk factors with incident dementia using a life-course perspective. The prevalence of modifiable risk factors for dementia differed by sex/gender and SES. No overall sex/gender difference in dementia risk was found. Dementia risk was higher among those who experienced childhood deprivation and those with lower occupational attainment and wealth. Though different associations between the modifiable risk factors and incident dementia were found among the subgroups, we only found a potential sex/gender difference in dementia risk for low cognitive activity, suggesting an increased dementia risk for women compared to men with low cognitive activity. No other sex/gender or consistent SES differences in the associations of modifiable risk factors for dementia and incident dementia were found, suggesting similar contributions of the modifiable risk factors to dementia risk among the subgroups.

In line with recent studies, the incidence of dementia in our study was similar among women and men^3^. The absence of a sex/gender difference in incident dementia compared to older studies that reported sex/gender differences in dementia risk might have been the result of a decrease in sex/gender differences in education due to the increase in educational opportunities. However, we still found a higher prevalence of low education in women compared to men, while in cohorts currently entering older ages there is evidence for a reversal of the gender gap, in that women reach higher levels of education compared to men in most Western and many non-Western countries^34^. Bloomberg et al.^35^ found better fluency scores for men compared to women among individuals with a low education in earlier birth cohorts, but women had better fluency scores than men among individuals with high education in later birth cohorts. This adds evidence for increases in cognitive reserve among women compared to the past^36–38^.

Overall, modifiable risk factors for dementia showed similar patterns of association with incident dementia among women and men. We observed a stronger association between low cognitive activity and incident dementia in women compared to men. Though this interaction became non-significant after correction for multiple testing, it may be that cognitive activity outside work is more important for building up cognitive reserve for women than for men. Traditionally, men are higher educated, have higher occupational positions, and work more hours and over more years compared to women^39^. Consequently, men may receive higher benefits through work in terms of cognitive stimulation, which makes cognitive stimulation outside work less important to build cognitive reserve for them. A recent systematic review indeed concluded that more cognitive reserve might be more beneficial in decreasing dementia risk among women, though more research is needed^40^. Evidence has shown that individuals who have a high compared to low cognitive stimulation at work have a decreased dementia risk after adjustment for other risk factors^41^. Furthermore, it was found that higher cognitive stimulation at work was associated with lower levels of proteins that inhibit central nervous system axonogenesis and synaptogenesis; in turn these proteins were associated with an increased risk of developing dementia^41^. Alternatively, the stronger association of cognitive activity with dementia risk for women compared to men in our study might have been a chance finding, as confidence intervals of the sex-specific estimates overlap. This should be in line with a previous study that did not find modification of sex/gender differences on the association of cognitive reserve with dementia risk^42^.

Other modifiable risk factors for dementia were of equal importance in women and men. Diabetes was associated with an increased risk for dementia, similarly in women and men. This is in line with data from the UK Biobank^12^ and a meta-analysis that found a 1.6 times increased dementia risk among individuals with type 2 diabetes in both sexes/genders^43^. The increased dementia risk for individuals with diabetes is important, as the worldwide obesity pandemic may lead to an accelerated increase in the prevalence of type 2 diabetes^44,45^. Screening for cognitive impairment in older individuals with diabetes and tailored glucose-lowering treatment is recommended^46^. Furthermore, mechanisms underlying dementia and type 2 diabetes overlap^47^. Intervention strategies focused on the modification of these shared risk factors may therefore have a positive effect on both conditions simultaneously.

In contrast to a recent UK Biobank study^12^, we did not observe sex/gender differences in the associations of obesity and hypertension with incident dementia. The UK Biobank has a substantial larger sample size (*n* >  = 464,616), which represents a larger statistical power to find sex/gender differences in cardiovascular and metabolic risk factors for dementia. However, the suggested increased dementia risk for obese women and decreased dementia risk for obese men in the UK Biobank were non-significant^12^. Furthermore, in ELSA dementia is based on self-reported or informant-reported physician-diagnosis or the IQCODE questionnaire, while the UK Biobank dementia only used hospital records and death certificates until recently. This might have resulted in more severe dementia cases in the UK Biobank compared to ELSA. Previous studies that investigated sex/gender differences in the association between blood pressure and incident dementia have reported mixed results^12,48–51^, which again might have been the result of heterogeneity among the studies. One study focused on midlife hypertension^48^, while two studies focused on specific types of dementia^49,51^. Furthermore, the number of participants who developed vascular dementia in one study was small (*n* = 47), which makes the results less reliable^49^. Gong et al. found an *U*-shaped relation between systolic blood pressure and incident dementia among men and a positive dose–response relationship among women using data of the UK Biobank^12^. As their confidence intervals for hypertension overlap with the confidence intervals in the current study, the absence of an association between hypertension and dementia risk cannot be excluded.

More studies that investigate sex/gender differences in behavioral risk factors for dementia, like social and cognitive activity, are recommended to replicate our findings in large population-based samples. Sindi et al.^11^ did not find associations between the proposed modifiable risk factors for dementia and incident dementia in men during a follow-up period of 20 to 30 years. This might have been the result of the relatively small number of men included in the study (98 dementia cases) compared to the number of women (174 dementia cases). Their finding that physical activity is only a protective factor for dementia for women might therefore be a chance finding, which is supported by the relatively large confidence interval. In addition, similar contributions of risk factors were found during a shorter follow-up period between 3 and 9 years. For instance, hopelessness and cohabiting were associated with a decreased dementia risk among both sexes, which corresponds to our finding for a similar protective effect of depression and social contact in both sexes.

The finding that lower SES during childhood or adulthood is associated with an increased dementia risk, is consistent with previous research^14–17^. Childhood SES may lead to an increased dementia risk through several pathways. One possible pathway is a chain of events starting with fetal malnutrition, followed by low birthweight, low childhood intelligence, low education, low occupation, and a unhealthy life style which predispose to many age-related diseases^52^. Fetal malnutrition may also affect physiological pathways directly, influencing neurodevelopment and blood pressure and glucose metabolism regulation, the later predisposing to obesity^52^. Indeed, low childhood SES has been related to smaller hippocampal volumes in late-life after adjustment for childhood mental ability, adulthood SES, sex, and education^53^. Childhood SES may also contribute to cognitive impairment through personality traits^54^. More research is needed to unravel the pathways from SES leading to dementia using a life-course approach.

Despite prevalence of heart disease being higher among the lower SES groups, an increased dementia risk for heart disease was found for those without childhood deprivation and those with high occupational attainment. However, these interactions were not confirmed in interactions with respectively parental occupation and wealth, and the interaction of childhood deprivation with heart disease became non-significant after correction for multiple testing. In addition, the association between heart disease and incident dementia was stronger and significant for individuals with a low parental occupation compared to those with a high parental occupation. The relatively small number of participants who developed dementia with heart disease (*n* = 77) resulted in estimates with large confidence intervals in the stratified analyses. Consequently, the numbers within the cells may have been too small to allow testing for interactions. Therefore, the increased dementia risk for heart disease among those with a high occupation need to be interpreted with caution and is likely a chance finding. We did not find other differences in exposure-outcome relations, suggesting a similar contribution of the modifiable risk factors to dementia among the subgroups.

While it is difficult to intervene on contextual socioeconomic conditions, inequalities in dementia risk profiles can be overcome through public policies that offer population-based healthcare and prevention to more vulnerable individuals. The World Health Organization proposed a global action plan on the public health response to dementia for the period 2017–2025^55^. Dementia should be included in other programs, policies, and campaigns on noncommunicable disease risk reduction and health promotion, by promoting physical activity, healthy nutrition, education, cognitive activity, and social engagement. Furthermore, tobacco use and excessive alcohol consumption should be discouraged. Population-based approaches are likely to be the most impactful, cost-effective, and meaningful to reduce the global burden of dementia. If carried out well, these can tackle inequality directly, are far-reaching, robust to changing understandings of dementia, and are likely to reduce the overall burden of chronic diseases in later life^56^.

Strengths of our study include its large sample size and population-based design; an extended follow-up period with biennial assessments; the use of several proxies for SES; the retrospective assessment of childhood SES, which make it possible to investigate associations with SES over the life course; and the extensive assessment of modifiable dementia risk factors. This study also has some limitations. First, the longitudinal population-based design comes along with nonresponse, selection and attrition bias^57^. This may have led to a healthier study sample and underestimated effects. Second, dementia is still underdiagnosed^58^. Incidence rates of dementia might therefore have been underestimated; however, we used an informant-based measure to detect (unreported) probable dementia. Third, dementia diagnosis, and some modifiable dementia risk factors were partly based on self-reported data and not clinical assessments. Fourth, within ELSA no distinction was made between specific dementia subtypes, which prevented us to test whether sex/gender and SES differences in modifiable risk factors differ by dementia subtype. Though, autopsy studies suggest most dementias to be of mixed pathology^59^. Fifth, there might have been reversed causality in which dementia risk factors are consequences of the preclinical phase of dementia instead of risk factors^60^. Sixth, despite the large study sample, some analyses (e.g., heart disease, smoking) might still have been underpowered. Lastly, in sensitivity analyses we corrected for multiple testing. The increased dementia risk for women with cognitive inactivity compared to men with cognitive inactivity became non-significant. Correction for multiple testing remains a point of discussion, and therefore, in agreement with current recommendations for observational studies^61^, we underscore the importance of replication of our results in future studies.

In conclusion, although no overall sex/gender differences in dementia risk were found, cognitive inactivity might be a more important dementia risk factor for women compared to men. Furthermore, dementia risk was higher among those who experienced childhood deprivation and those with low adulthood SES. No consistent socioeconomic difference in modifiable dementia risk was found, suggesting modifiable risk factors to contribute similarly to dementia risk among subgroups with different childhood and adulthood SES. Our findings suggest that sex/gender and socioeconomic inequalities in dementia risk are likely mainly driven by differences in the prevalence of modifiable risk factors for dementia. Inequalities in the prevalence of modifiable risk factors may be tackled through public policies that offer population-based healthcare and prevention to more vulnerable individuals.

## Supplementary Information

Supplementary Information.

## Data Availability

The English Longitudinal Study of Ageing (ELSA) was developed by a team of researchers based at University College London, the Institute for Fiscal Studies and the National Centre for Social Research. The data are linked to the UK Data Archive and freely available through the UK data services and can be accessed here: https://discover.ukdataservice.ac.uk.
