# Supplementary material for: Sex/gender and socioeconomic differences in modifiable risk factors for dementia
Source: Sci Rep. 2023 Jan 3;13:80. doi: 10.1038/s41598-022-27368-4 (PMC9810648; doi:10.1038/s41598-022-27368-4)
Supplement: Supplementary file 1 — Supplementary Information. [file 41598_2022_27368_MOESM1_ESM.pdf]

Supplementary Material

Sex/gender and socioeconomic differences in modifiable risk factors for dementia

Anouk F.J. Geraets, Ph.D.[1] & Anja K. Leist, Ph.D.[1]

[1]Department of Social Sciences, University of Luxembourg, Esch-Sur-Alzette, Luxembourg

eTable 1. Strategies to reduce missing values (if information at Wave 4 was missing).

Variable	Strategies to reduce missing values
**Socioeconomic factors**	
Occupation	- Look at identical information from ELSA Wave 2-6, 8 and 9 taking longitudinal weight into account
Wealth	- Look at identical information from ELSA Wave 1-9 taking longitudinal weight into account
**Dementia risk factors**	
Heart disease	- Look at self-report ELSA Wave 1-4 for ever reported heart disease and Wave 5-9 for never reported heart disease
Hypertension	- Look at self-report and medication use from ELSA Wave 5 and Wave 3
Hypercholesterolemia	- Look at self-report and medication use from ELSA Wave 5 and Wave 3
Diabetes	- Look at self-report at ELSA Wave 2 and 3 for ever reported diabetes and Wave 5-9 for never reported diabetes type 2
Obesity	- Look at identical information from ELSA Wave 6
Hearing impairment	- Look at identical information from ELSA Wave 5 and Wave 3
Smoking	- Look at identical information from ELSA Wave 5 and Wave 3
High alcohol consumption	- Look at identical information from ELSA Wave 5
Physical inactivity	- Look at identical information from ELSA Wave 5 and Wave 3
Sleep disturbance	- Look at identical information from ELSA Wave 6
Unhealthy diet	- Look at identical information from ELSA Wave 5 and Wave 3
Depression	- Look at identical information from ELSA Wave 5 and Wave 3
Low educational attainment	- Look at identical information from ELSA Wave 5-9 (when information from Wave 4 and previous Waves was not available)
Low social contact	- Look at identical information from ELSA Wave 5 and Wave 3
Low cognitive activity	- Look at identical information from ELSA Wave 5 and Wave 3

ELSA indicates English Longitudinal Study of Ageing.

eTable 2. **Operationalization of socioeconomic inequalities and modifiable risk factors for dementia**

Factor	Operationalization
**Socioeconomic factors**	
Childhood deprivation	Self-reported childhood deprivation was defined as having one or more of the following deprivations: 1) having none or few books (0–10 books) in the home; 2) overcrowding in the home (more than two persons per bedroom); and 3) having no indoor toilet, no fixed bath, no central heating, and/or no hot and cold running water supply to the home.
Occupation breadwinner	Self-reported occupational attainment of the breadwinner during childhood was classified as highly skilled (managers, senior officials, business owners, professionals, and technicians) versus lower-skilled (excluding armed forces, other jobs, something else, and retired) based on the International Labor Organization's definition of skill level [1].
Occupation	Self-reported occupational attainment was based on the U.K. National Statistics Socioeconomic Classification (NS-SEC) system [2] and grouped into routine/manual, intermediate, and managerial/professional.
Wealth	Self-reported household wealth was calculated by summing wealth from the total value of a respondent's home (minus outstanding mortgage payments), physical wealth (e.g., jewelry), business assets (e.g., investments), and financial assets such as cash and savings (minus debts and loans). The overall measure of wealth was divided into tertiles: low, medium, and high.
**Dementia risk factors**	
Heart disease	Self-reported diagnosis of angina pectoris or myocardial infarction.
Hypertension	Self-reported diagnosis, mean systolic blood pressure ≥140 mm Hg or mean diastolic blood pressure ≥90 mm Hg [3] or use of blood pressure lowering medication.
Hypercholesterolemia	Self-reported diagnosis, total cholesterol level of ≥5.0 mmol/L and low-density lipoprotein of ≥3.0 mmol/L, following the guidelines of the National Health Service UK [4] or use of lipid-modifying medication.
Diabetes	Self-reported diagnosis, blood glycated hemoglobin level ≥ 48 mmol/mol (6.5%) according to the WHO guidelines [5] or use of glucose lowering medication.
Obesity	Established cut-offs according to the WHO guidelines [6]. Waist circumference (men: > 102 cm; women: > 88 cm) and waist-to-hip ratio (men: >90; women: >85) were only used if data on body mass index (BMI ≥ 30) was missing.
Hearing impairment	Self-reported hearing impairment (while using hearing aid if appropriate) was defined as absent (excellent/very good/good hearing or present (fair/poor hearing).
Smoking	Self-reported current smokers or non-smokers.
High alcohol consumption	Self-reported frequency of any alcohol consumed in the past 12 months. High alcohol use was defined as >14 glasses per week according to recent UK alcohol guidelines [7]
Physical inactivity	Self-reported engagement in vigorous, moderate, or mild physical activity during leisure time (more than once per week, once per week, one to three times per month, hardly ever). Participants were dichotomized into physically active (≥1/week) or physically inactive (1-3 times/month, hardly ever/never).
Sleep disturbance	One or more self-reported sleep problems three or more times a week during the last month: 1) difficulties falling asleep, 2) waking up several times a night,

	and 3) waking up after their usual amount of sleep feeling tired and worn out [8].
Unhealthy diet	Reported number of fruits and vegetables consumed by the participant the previous day. An unhealthy diet was defined as consuming four or less portions of fruits and vegetables on a daily basis [9]
Depression	Self-reported diagnosis and/or CES-D 8 score ≥3 [10]
Low educational attainment	Self-reported educational attainment was grouped into two categories: low (no formal qualifications) and medium/high (National Vocational Qualifications level 1 till degree level).
Low social contact	Self-reported social contact undertaken in the last 12 months (e.g., being a member of any organizations, clubs, or societies). Engagement in none of these contacts was considered as low social activity (lowest tertile).
Low cognitive activity	Self-reported engagement in intellectual activities undertaken in the last 12 months (e.g., read the newspaper daily, have a hobby, take a holiday, using the internet). Engagement in two or less of these activities was considered as low cognitive activity (lowest tertile).

WHO indicates World Health Organization; BMI, body mass index; JSS, Jenkins Sleep Scale; CES-D 8, Center for Epidemiologic Studies Depression Scale; UK, United Kingdom.

eTable 3. Characteristics study population stratified by sex/gender

	Women (n=4,935)	Men (n=4,006)	*p*-value
**Dementia**			
Incident dementia cases, n (%)	242 (4.9)	154 (3.8)	0.015
**Demographics**			
Age, mean (standard deviation)	66.3 (10.0)	65.8 (9.5)	0.038
**Socioeconomic factors**			
Childhood deprivation, n (%)	1,273 (40.6)	1,052 (42.1)	0.252
Low/medium occupation breadwinner, n (%)	1,462 (61.8)	1,180 (64.8)	0.050
Occupational attainment, n (%)			<0.001
Low	2,008 (41.7)	1,500 (37.8)	
Medium	1,413 (29.3)	838 (21.1)	
High	1,397 (29.0)	1,631 (41.1)	
Wealth, n (%)			<0.001
Low	1,703 (34.5)	1,227 (30.6)	
Medium	1,671 (33.9)	1,376 (34.4)	
High	1,561 (31.6)	1,403 (35.0)	
**Dementia risk factors**			
Heart disease, n (%)	418 (8.5)	538 (13.4)	<0.001
Hypertension, n (%)	3,059 (63.3)	2,583 (65.6)	0.024
Hypercholesterolemia, n (%)	2,932 (61.3)	2,152 (55.4)	<0.001
Diabetes, n (%)	1,149 (23.3)	1,008 (25.2)	0.039
Obesity, n (%)	1,569 (35.1)	1,099 (30.0)	<0.001
Hearing impairment, n (%)	768 (15.6)	1,012 (25.3)	<0.001
Smoking, n (%)	686 (14.0)	537 (13.5)	0.520
High alcohol consumption, n (%)	2,218 (47.8)	1,602 (42.7)	<0.001
Physical inactivity, n (%)	1,690 (34.3)	959 (23.9)	<0.001
Sleep disturbance, n (%)	2,941 (60.3)	2,024 (51.3)	<0.001
Unhealthy diet, n (%)	1,880 (39.6)	1,645 (42.7)	0.004
Depression, n (%)	1,274 (26.1)	609 (15.3)	<0.001
Low educational attainment, n (%)	1,618 (36.6)	939 (24.6)	<0.001
Low social contact, n (%)	1,484 (31.5)	1,052 (27.4)	<0.001
Low cognitive activity, n (%)	1,669 (34.1)	1,190 (30.0)	<0.001

P-values are presented for the comparison of women with men (independent samples t-tests, Mann-Whitney U-tests, and χ^2-tests).

eTable 4. Characteristics study population stratified by wealth

	Low (n=2,930)	Medium (n=3,047)	High (n=2,964)
**Dementia**			
Incident dementia cases, n (%)	167 (5.7)	129 (4.2)	100 (3.4)
**Demographics**			
Age, mean (standard deviation)	66.7 (10.6)	66.1 (9.5)	65.5 (9.2)
Women, n (%)	1,703 (58.1)	1,671 (54.8)	1,561 (52.7)
**Dementia risk factors**			
Heart disease, n (%)	421 (14.4)	309 (10.1)	226 (7.6)
Hypertension, n (%)	2,051 (71.3)	1,894 (63.3)	1,697 (58.4)
Hypercholesterolemia, n (%)	1,606 (56.6)	1,732 (58.6)	1,746 (60.7)
Diabetes, n (%)	865 (29.5)	723 (23.7)	569 (19.2)
Obesity, n (%)	1,028 (39.3)	925 (33.4)	715 (26.0)
Hearing impairment, n (%)	715 (24.4)	593 (19.5)	472 (15.9)
Smoking, n (%)	671 (23.0)	345 (11.4)	207 (7.0)
High alcohol consumption, n (%)	1,516 (57.0)	1,239 (42.9)	1,065 (37.5)
Physical inactivity, n (%)	1,239 (42.3)	841 (27.6)	569 (19.2)
Sleep disturbance, n (%)	1,821 (63.0)	1,677 (55.8)	1,467 (50.1)
Unhealthy diet, n (%)	1,368 (49.4)	1,211 (41.0)	946 (32.8)
Depression, n (%)	898 (30.9)	567 (18.8)	418 (14.2)
Low educational attainment, n (%)	1,312 (48.4)	843 (30.3)	402 (14.6)
Low social contact, n (%)	1,161 (42.5)	857 (29.2)	518 (18.0)
Low cognitive activity, n (%)	1,428 (49.5)	869 (28.7)	562 (19.1)

eTable 5. Characteristics of individuals included and excluded in the study

	Included (n=8,941)	Excluded (n=752)	*p-value*
**Dementia**			
Incident dementia cases, n (%)	396 (4.4)	14 (1.9)	0.001
**Demographics**			
Age, mean (standard deviation)	66.1 (9.8)	66.0 (10.5)	0.801
Women, n (%)	4,935 (55.2)	426 (56.7)	0.441
**Socioeconomic factors**			
Childhood deprivation, n (%)	2,325 (41.2)	142 (49.0)	0.009
Low/medium occupation breadwinner, n (%)	2,642 (63.1)	1 (100)	0.444
Occupation, n (%)			0.001
Low	3,508 (39.9)	322 (46.6)	
Medium	2,251 (25.6)	167 (23.5)	
High	3,028 (34.5)	213 (29.9)	
Wealth, n (%)			<0.001
Low	2,930 (32.8)	321 (42.7)	
Medium	3,047 (34.1)	229 (30.5)	
High	2,964 (33.2)	202 (26.9)	
**Dementia risk factors**			
Heart disease, n (%)	956 (10.7)	81 (10.8)	0.908
Hypertension, n (%)	5,642 (64.3)	581 (78.0)	<0.001
Hypercholesterolemia, n (%)	5,084 (58.6)	294 (52.6)	0.005
Diabetes, n (%)	2,157 (24.1)	359 (47.7)	<0.001
Obesity, n (%)	2,668 (32.8)	133 (31.2)	0.507
Hearing impairment, n (%)	1,780 (19.9)	173 (23.1)	0.037
Smoking, n (%)	1,223 (13.7)	126 (16.9)	0.016
High alcohol consumption, n (%)	3,820 (45.5)	264 (52.4)	0.003
Physical inactivity, n (%)	2,649 (29.6)	306 (40.8)	<0.001
Sleep disturbance, n (%)	4,965 (56.3)	403 (56.3)	0.993
Unhealthy diet, n (%)	3,525 (41.0)	277 (48.1)	0.001
Depression, n (%)	1,883 (21.3)	206 (28.5)	<0.001
Low educational attainment, n (%)	2,557 (31.0)	286 (42.1)	<0.001
Low social contact, n (%)	2,536 (29.7)	203 (36.1)	0.001
Low cognitive activity, n (%)	2,859 (32.3)	218 (36.5)	0.034

Dementia cases up to Wave 4 is n=193. *P*-values are presented for the comparison of individuals excluded with individuals included in the analytic sample (independent samples t-tests, Mann-Whitney U-tests, and χ^2-tests).

References

1 International Standard Classification of Occupations (ISCO). (ILOSTAT, 2008).

2 Galobardes, B., Shaw, M., Lawlor, D. A. & Lynch, J. W. Indicators of socioeconomic position (part 2). *Journal of epidemiology and community health* **60**, 95 (2006). https://doi.org:10.1136/jech.2004.028092

3 Chalmers, J. *et al.* 1999 World Health Organization-International Society of Hypertension Guidelines for the management of hypertension. Guidelines sub-committee of the World Health Organization. *Clinical and experimental hypertension (New York, NY: 1993)* **21**, 1009-1060 (1999). https://doi.org:10.3109/10641969909061028

4 Cholesterol levels. (National Health Service 2022).

5 Use of glycated haemoglobin (HbA1c) in diagnosis of diabetes mellitus: abbreviated report of a WHO consultation. (World Health Organization, 2011).

6 Waist circumference and waist-hip ratio: report of a WHO expert consultation, Geneva, 8-11 December 2008. (World Health Organization, 2011).

7 UK Chief Medical Officers' alcohol guidelines review: Summary of the proposed new guidelines. (UK Department of Health, 2016).

8 Jenkins, C. D., Stanton, B.-A., Niemcryk, S. J. & Rose, R. M. A scale for the estimation of sleep problems in clinical research. *Journal of clinical epidemiology* **41**, 313-321 (1988).

9 5 A Day. (National Health Service London, 2009).

10 Turvey, C. L., Wallace, R. B. & Herzog, R. A revised CES-D measure of depressive symptoms and a DSM-based measure of major depressive episodes in the elderly. *International psychogeriatrics* **11**, 139-148 (1999). https://doi.org:10.1017/S1041610299005694
